# Sex Differences in Cardiovascular Risk Associated With Long-Term PM_2.5_ Exposure: A Systematic Review and Meta-Analysis of Cohort Studies

**DOI:** 10.3389/fpubh.2022.802167

**Published:** 2022-02-02

**Authors:** Jia Zhang, Xinyan Wang, Mengfan Yan, Anqi Shan, Chao Wang, Xueli Yang, Naijun Tang

**Affiliations:** ^1^Institute of Epidemiology and Health Statistics, School of Public Health, Lanzhou University, Lanzhou, China; ^2^Department of Occupational and Environmental Health, School of Public Health, Tianjin Medical University, Tianjin, China; ^3^Center for Reproductive Medicine, Tianjin Central Hospital of Obstetrics and Gynecology, Tianjin, China; ^4^Tianjin Key Laboratory of Environment, Nutrition and Public Health, Tianjin, China; ^5^Department of Epidemiology and Biostatistics, Beijing Research Institute of Traumatology and Orthopaedics, Beijing Jishuitan Hospital, Beijing, China; ^6^Department of Molecular Orthopaedics, Beijing Research Institute of Traumatology and Orthopaedics, Beijing Jishuitan Hospital, Beijing, China

**Keywords:** air pollution, meta-analysis, cohort, cardiovascular diseases, sex differences

## Abstract

**Background:**

Established evidence suggests risks of developing cardiovascular disease are different by sex. However, it remains unclear whether associations of PM_2.5_ with cardiovascular risk are comparable between women and men. The meta-analysis aimed to examine sex differences in associations of ischemic heart disease (IHD) and stroke with long-term PM_2.5_ exposure.

**Methods:**

PubMed, EMBASE and Cochrane Library were searched until May 2, 2021. We included cohort studies reporting sex-specific associations of long-term PM_2.5_ exposure (e.g., ≥1 year) with IHD and stroke. The primary analysis was to estimate relative risk (RR) of PM_2.5_-outcome in women and men separately, and the additional women-to-men ratio of RR (RRR) was explored to compare sex differences, using random-effect models.

**Results:**

We identified 25 eligible studies with 3.6 million IHD and 1.3 million stroke cases among 63.7 million participants. A higher level of PM_2.5_ exposure was significantly associated with increased risk of IHD in both women (RR = 1.21; 95% CI, 1.15–1.27) and men (RR = 1.12; 95% CI, 1.07–1.17). The women-to-men RRR of IHD was 1.05 (95% CI, 1.02–1.08) per 10 μg/m^3^ increment in PM_2.5_ exposure, indicating significant excess risk of IHD in women. The significant risks of stroke associated with PM_2.5_ were obtained in both women (RR = 1.11; 95% CI, 1.08–1.13) and men (RR = 1.11; 95% CI, 1.07–1.14), but no significant women-to-men RRR was observed in stroke (RRR = 1.00; 95% CI, 0.96–1.04).

**Conclusions:**

The study identified excess risk of IHD associated with long-term PM_2.5_ exposure in women. The findings would not only have repercussions on efforts to precisely evaluate the burden of IHD attributable to PM_2.5_, but would also provide novel clues for cardiovascular risk prevention accounting for sex-based differences.

## Introduction

Cardiovascular disease (CVD) is the leading cause of the global disease burden (1), and accumulating evidence highlighted that sex differences existed in the risk factors, manifestation, and treatment of the cardiovascular clinical spectrum (2, 3). Meta-analyses have illustrated that several factors (e.g., smoking and diabetes) had significantly greater cardiovascular risk in women than men (4, 5).

In addition, established evidence identified a relationship of cardiovascular morbidity linked to long-term exposure to PM_2.5_ (i.e., particulate matter <2.5 μm in diameter) (6). However, there is a debate on sex-based discrepancies for the PM_2.5_-CVD associations. Several studies observed higher risks of CVD associated with PM_2.5_ in women (7–9), while others reported similar effect estimations between sexes (10–12). To our knowledge, there is no quantitative synthesis of published literature, comparing sex differences in the relationship between long-term PM_2.5_ exposure and CVD. A comprehensive investigation of potential sex differences in PM_2.5_-related risk of CVD would extend our understanding of deleterious effects due to air pollution. If the different cardiovascular risks associated with long-term exposure to PM_2.5_ could be confirmed in women and men, it would have implications for precise assessment of disease burden attributable to PM_2.5_ exposure. Meanwhile, it could also provide novel clues for cardiovascular risk prevention, accounting for sex-based differences.

In this study, considering various types of CVD, ischemic heart disease (IHD) and stroke were selected as two main endpoints since they have been the top leading causes of CVD burden (1) and mostly reported by previous original studies on associations between PM_2.5_ and CVD (13). Herein, we conducted a meta-analysis of cohort studies to examine sex-specific risks of long-term exposure to PM_2.5_ with incident IHD and stroke, and further to identify whether a more detrimental association of PM_2.5_ exposure might exist in women, using the pooled estimations of relative risk ratio between women and men.

## Methods

### Search Strategy

This study was conducted following the Preferred Reporting Items for Systematic Reviews and Meta-Analyses (PRISMA) guidelines with a checklist in Supplementary Table 1 (14). Briefly, we systematically searched the relevant articles in PubMed, EMBASE and Cochrane Library until May 2^nd^, 2021. Search terms included PM_2.5_ exposure, cardiovascular outcome, and study design, including keywords as follows: (1) particulate matter: PM_2.5_, fine particulate matter, (2) cardiovascular outcome: cardiovascular disease, cardiovascular event, stroke, cerebrovascular disease, myocardial ischemia, coronary artery disease, heart failure, myocardial infarction, ischemic heart disease, angina pectoris, coronary heart disease, heart attack, acute coronary syndrome, and (3) cohort study: cohort, longitudinal study, longitudinal, odds ratio, relative risk, hazard ratio. The full electronic search strategies for each database are shown in Supplementary Tables 2–4. Additionally, we manually checked the relevant reviews and references of included studies to complement articles.

### Study Selection

Two authors (J.Z. and X.W.) independently screened the titles and abstracts, and then full texts of the potential qualified studies were further assessed. A third reviewer (M. Y.) would check the article and make a decision if there was any disagreement. Studies eligible for inclusion met the following conditions: (1) Participants: general human population with ambient PM_2.5_ exposure, excluding those with workplace exposure to PM_2.5_; (2) Exposures: the exposure of interest included long-term exposure (i.e., ≥1 year) to PM_2.5_; (3) Comparisons: studies provided sex-specific effect estimates of the PM_2.5_-outcome association with relative risk (RR) or hazard ratio (HR) as well as their 95% confidence intervals (CIs) per 10-μg/m^3^ increment of PM_2.5_ exposure; (4) Outcomes: study outcomes included at least either IHD or stroke; (5) Designs: studies were restricted to cohort design. Those excluded studies were: (1) reviews or animal experiments; (2) targeting short-term exposures or acute effects; (3) irrelevant research outcomes; (4) with other study designs (e.g., ecological studies, cross-sectional studies, or case-control studies, etc.); (5) cohorts among patients with specific diseases; (6) studies unavailable to explicit sex-subgroup results; (7) studies from the same cohort with overlapping participants. When multiple articles examined the same outcome based on the same cohort, only one study per cohort was included with the longest duration of follow-up or the most recent published article.

### Data Extraction and Quality Assessment

Two investigators (J.Z. and X.W.) independently extracted data on author name, publication year, country, study name, study period, population characteristics, sample size, methods of PM_2.5_ exposure measurement, International Classification of Diseases (ICD) codes of outcomes, number of cases, covariates adjusted in the statistical model, and sex-specific effect estimates (HRs or RRs with 95% CIs). When a study reported multiple results using regression models with different covariates, the result of fully adjusted model was chosen. The quality of included studies was evaluated using Newcastle-Ottawa Assessment Scale (NOS) (15). Briefly, the NOS is based on eight items from three main aspects: (1) Selection of study population; (2) Comparability of cohorts; (3) Assessment of outcomes and adequacy of follow up of cohorts. There were four, one, and three items for the categories of Selection, Comparability, and Outcome, respectively. Each study could be awarded a maximum of one point for each numbered item within the Selection and Outcome categories, but a maximum of two points could be given for Comparability (Supplementary Table 5). The total score of NOS ranged from 0 to 9, and studies with 7 or more were considered as high-quality in the meta-analysis.

### Statistical Analysis

In this meta-analysis, the major endpoints were incident risks of IHD and stroke. The primary estimates were the pooled sex-specific relative risk (RR) and the women-to-men ratio of RR (RRR) per 10-μg/m^3^ increment of PM_2.5_ exposure. The women-to-men RRRs with 95% CIs have been commonly used to assess the excessive risk for exposure-disease association in women compared to men (4, 5).

For each study, sex-specific RRs and 95% CIs were used for associations between cardiovascular outcomes and long-term PM_2.5_ exposure. Hazard Ratios (HRs) were considered equivalent to RRs. All the effect estimates with 95% CIs were converted to a comparable unit of 10 μg/m^3^. The pooled RR for women or men was separately obtained using the random-effect model by the method of DerSimonian and Laird, prior accounting for between-study heterogeneity (16).

Furthermore, the sex-specific RR was log-transformed, and the women-to-men difference in log-RRs was computed within each study. The differences were then pooled across studies using random-effect meta-analysis weighted by the inverse variances of the log-RRs, and finally back-transformed to the raw scale, obtaining the pooled women-to-men RRR. The standard error of the log RRR was derived from the sum of variance of the sex-specific log-RR for each study, followed by taking the square root. The details of the identical approach have been described elsewhere (4, 5).

Heterogeneity of between-study was tested by the coefficient of inconsistency (*I*^2^ statistic). Sensitivity analysis by excluding one study at a time was conducted, and publication bias was graphically examined using funnel plots along with the Begg's test. Stata version 12.0 software (StataCorp, TX) was used for all meta-analyses. All *P*-values were two-sided with a significant level at 0.05.

## Results

### Article Selection and Description

**Figure 1:**
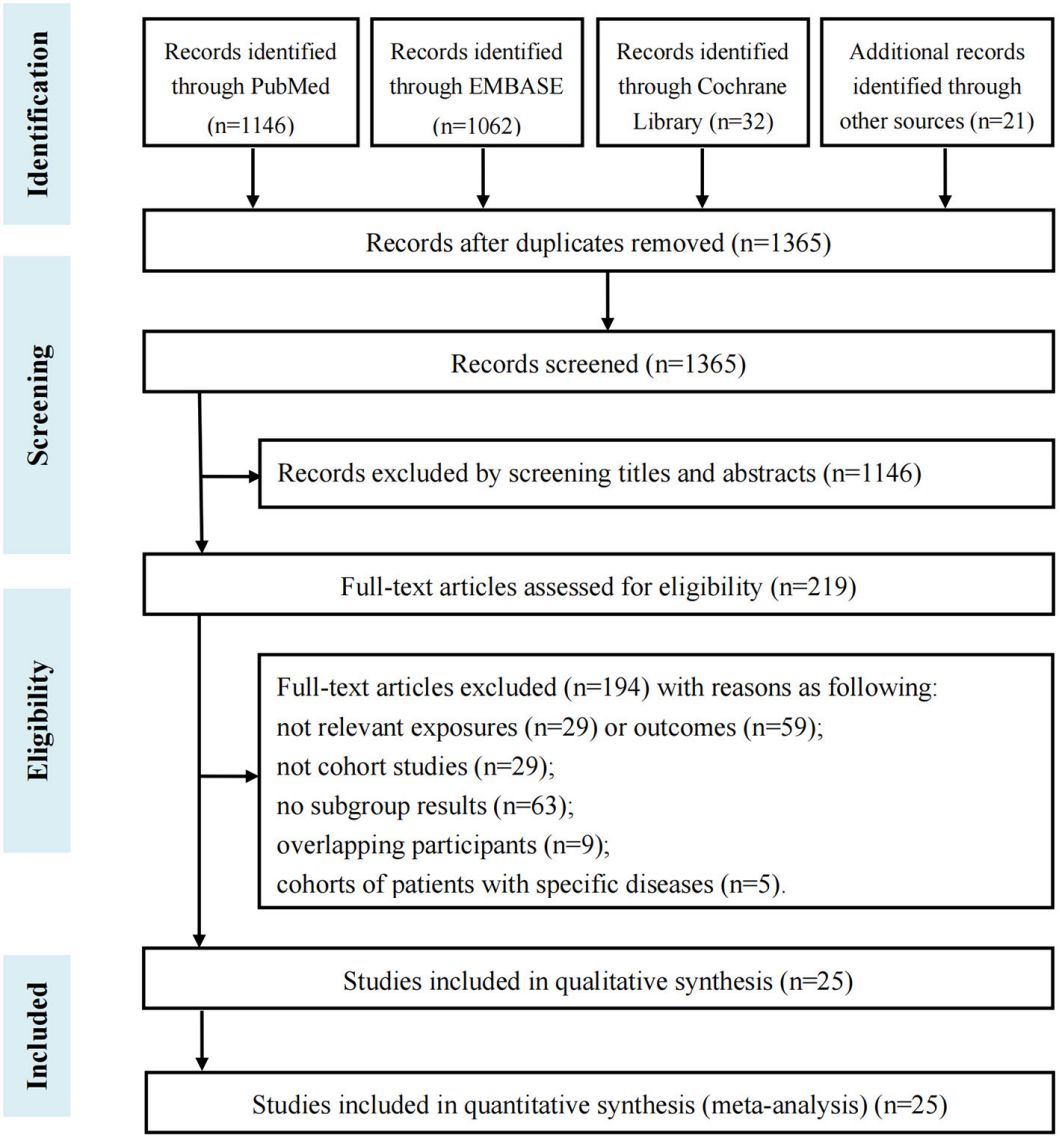
Flowchart of study inclusion and exclusion.

The flowchart of study inclusion and exclusion is shown in Figure 1. After screening 1,365 records, 219 articles were moved to the full-text review. Finally, there were 25 eligible publications among 67.3 million participants included in the further meta-analyses (7–12, 17–35), and the summarized characteristics of studies are shown in Table 1. Of the 25 eligible articles, 11 were based on cohorts from North America (7, 9, 10, 19–24, 26, 34), 5 from European countries (17, 18, 25, 28, 30), 6 from Asian populations (11, 12, 27, 31, 33, 35), 1 from Australia (29), and 2 from multiple countries (8, 32). Information on the quality assessment of studies is shown in Supplementary Table 5. After evaluating the design and description of the included studies using the NOS, it indicated 21 studies (84%) scored ≥7 as high-quality research. Furthermore, details of study characteristics for 17 publications on the outcome of IHD are listed in Supplementary Table 6 (7–12, 17–27), and characteristics of 18 articles on stroke are shown in Supplementary Table 7 (8, 9, 17, 19, 21–23, 25–35).

**Table 1:** Summary of 25 articles included in the meta-analyses.

**ID**	**First author (Publication year)**	**Country/ Region**	**Study name**	**Study period**	N^*^(Women %)	**Age range of participants**	**PM_**2.5**_, μg/m^**3**^ (mean or range)**	**Outcome types (Number of cases)**
1	Amini et al. (28)	Danish	DNC	1993–2014	23,423 (100%)	>44 years	19.7	Incidence: stroke (1,078)
2	Bai et al. (10)	Canada	ONPHEC	2001–2015	5,141,172 (52.3%)	35–85 years	9.6	Incidence: MI (197,628)
3	Cai et al. (17)	UK and Norway	HUNT, EPIC-Oxford, and UK Biobank	1993–2013	355,732 (58%)	≥20 years	9.9	Incidence: CVD (21,081); IHD (3,515); stroke (1,845)
4	Chen et al. (7)	USA	AHSMOG	1977–1998	3,239 (64.5%)	≥25 years	29	Mortality: IHD (250)
5	Cramer et al. (18)	Danish	DNC	1993–2014	22,882 (100%)	>44 years	19.6	Incidence: MI (641); mortality: MI (121)
6	Dirgawati et al. (29)	Australia	HIMS	1996–2012	11,627 (0%)	≥65 years	5.1	Incidence: stroke (1,453); mortality: stroke (325)
7	Elliott et al. (19)	USA	NHS	1988–2008	104,990 (100%)	30–55 years	13.7	Incidence: CVD (6,074); MI (3,304); stroke (2,848)
8	Gandini et al. (30)	Italy	ILS	2001–2008	74,989 (52.7%)	>35 years	10–30	Incidence: stroke (3,380)
9	Hart et al. (20)	USA	Trucking Industry Cohort	1985–2000	53,814 (0%)	15.3–84.9 years	14.1	Mortality: CVD (1,682); IHD (1,109)
10	Huang et al. (31)	China	China-PAR	1992–2015	117,575 (59%)	≥ 18 years	64.9	Incidence: stroke (3,540)
11	Hystad et al. (8)	21 countries	PURE study	2003–2018	157,436 (58%)	35–70 years	47.5	Incidence: CVD (9,152); MI (4,083); stroke (4,139); mortality: CVD (3,219)
12	Li et al. (11)	China	China-PAR	1992–2015	118,229 (58.9%)	≥ 18 years	64.96	Incidence: IHD (1,586); mortality: IHD (550)
13	Lin et al. (32)	6 countries	WHO SAGE	2007–2010	45,625 (56.8%)	≥ 18 years	23.09	Incidence: stroke (1,239)
14	Lipsett et al. (21)	USA	CTS	1995–2005	124,614 (100%)	22–104 years	15.64	Incidence: MI (722); stroke (969); mortality: CVD (1,630); IHD (773); stroke (382)
15	Miller et al. (22)	USA	WHI	1994–2000	58,610 (100%)	50–79 years	13.5	Incidence: CVD (1,816); IHD (1,268); stroke (600); MI (584) mortality: CVD (261); IHD (80); stroke (122)
16	Pinault et al. (23)	Canada	CanCHEC	2001–2011	2,448,500 (51.6%)	25–90 years	7.37	Mortality: IHD (52,200); stroke (22,000)
17	Puett et al. (24)	USA	Health Professionals Follow-Up Study cohort	1986–2003	17,545 (0%)	40–75 years	17.8	Incidence: MI (646); CVD (1,661); mortality: IHD (746)
18	Qiu et al. (33)	China	Elderly Hong Kong Residents cohort	1998–2010	61,447 (65.9%)	≥65 years	35.8	Incidence: stroke (6,733)
19	Shin et al. (34)	Canada	ONPHEC	2001–2015	5,071,956 (52%)	35–85 years	9.8	Incidence: stroke (122,545)
20	Stockfelt et al. (25)	Sweden	PPS cohort	1990–2011	5,850 (0%)	64–75 years	9.3	Incidence: IHD (1,826); stroke (1,139)
		Sweden	GOT-MONICA cohort	1990–2011	4,500 (52%)	25–64 years	8.5	Incidence: IHD (440); stroke (252)
21	Villeneuve et al. (26)	Canada	CNBSS	1980–2005	89,248 (100%)	40–59 years	9.1	mortality: CVD (1,845); IHD (903); stroke (434)
22	Wang et al. (9)	USA	Medicare Beneficiaries Cohort in US	2000–2008	52,954,845 (55.2%)	65–120 years	10.32	Mortality: CVD (6,371,713); IHD (3,323,527); stroke (1,147,050)
23	Wong et al. (12)	China	Elderly Hong Kong Residents cohort	1998–2011	59,591 (65%)	≥65 years	35.3	Mortality: CVD (4,656); IHD (1,810); stroke (1,621)
24	Yang et al. (35)	China	four cities cohort in northern China	1998–2009	38,140 (50.2%)	≥22 years	66.3	Mortality: stroke (254)
25	Yin et al. (27)	China	Chinese Male Cohort	1990–2006	189,793 (0%)	≥40 years	43.7	Mortality: CVD (18,859); IHD (3,752); stroke (11,301)

* *N, Number of participants*.

*AHSMOG, Adventist Health Study on the Health Effects of Smog; CanCHEC, Canadian Census Health and Environment Cohort; China-PAR project, Prediction for Atherosclerotic Cardiovascular Disease Risk in China; CNBSS, Canadian National Breast Screening Study; CTS, California Teachers Study; CVD, cardiovascular disease; DNC, Danish Nurse Cohort; EPIC-Oxford, European Prospective Investigation into Cancer and Nutrition; GOT-MONICA cohort, Multinational Monitoring of Trends and Determinants in Cardiovascular Diseases; HIMS, Health in Men Study; HUNT, Helseundersøkelsen i NordTrøndelag; IHD, ischemic heart disease; ILS, Italian Longitudinal Study; MI, myocardial infarction; NHS, Nurses' Health Study; ONPHEC, Ontario Population Health and Environment Cohort; PPS cohort, Primary Prevention Study cohort; PURE study, Prospective Urban and Rural Epidemiology study; WHI, Women's Health Initiative; WHO SAGE, World Health Organization Study on Global Ageing and Adult Health*.

### Risk of IHD Associated With PM_2.5_ in Women and Men

In the 17 studies on associations of long-term PM_2.5_ exposure with risk of IHD, 10 studies defined the outcome of IHD using the same codes of ICD (ICD-9: 410–414; ICD-10: I20-I25) (Supplementary Table 6). The outcome of 1 study focused on myocardial infarction (MI) (ICD-10: I21–I22) (8), and another 3 used a narrower definition of MI (ICD-8 and ICD-9: 410; ICD-10: I21) (10, 18, 19). Only 3 articles did not list ICD codes of their IHD descriptions (11, 22, 24). Based on scores of the NOS, 14 of the 17 included studies were high-quality.

**Figure 2:**
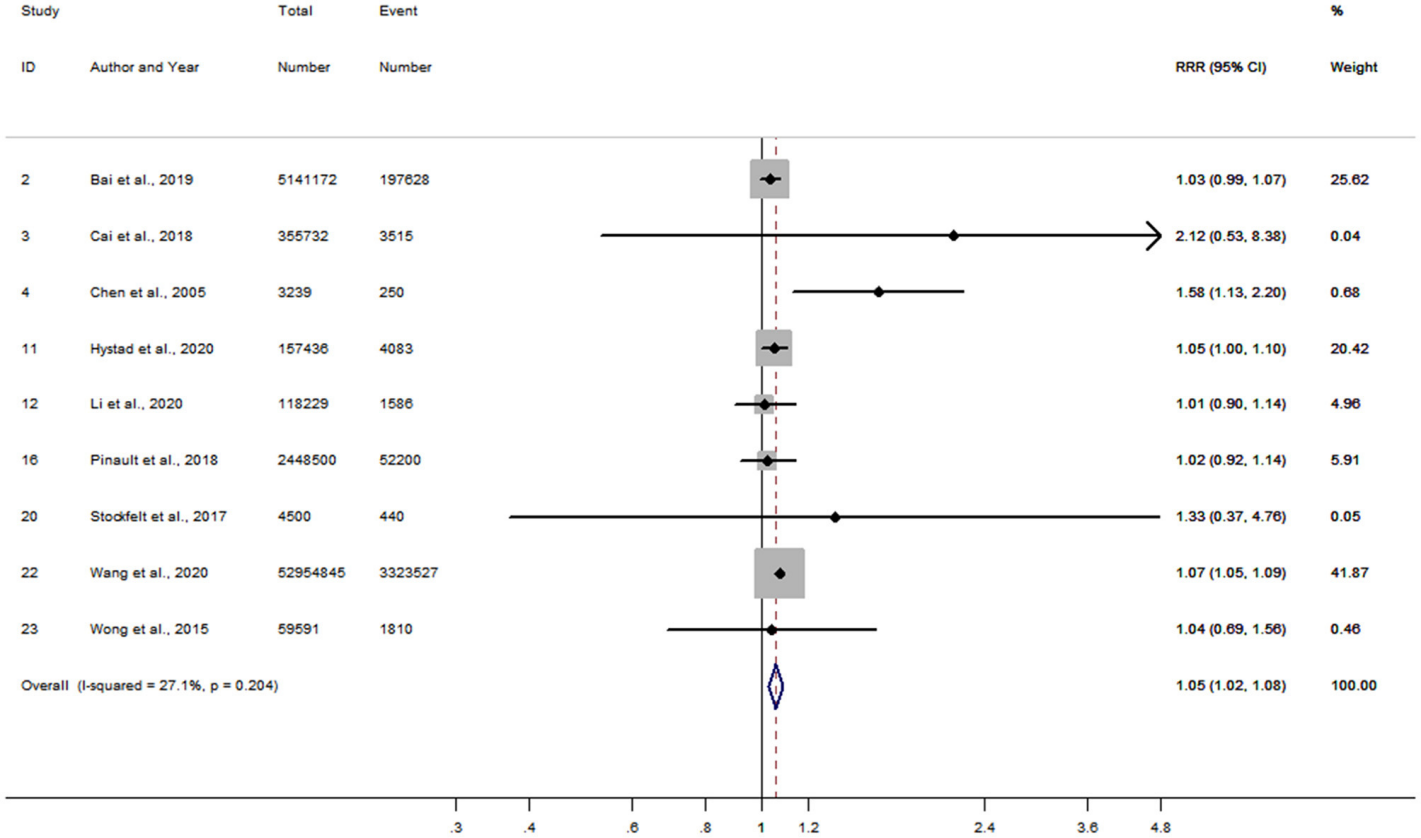
Forest plot for the women-to-men RRR of IHD per 10 μg/m^3^ increase in PM_2.5_ exposure. CI, confidence interval; IHD, ischemic heart disease; RRR, ratios of relative risk.

Of the 17 studies, 14 results in women were combined to obtain a RR of 1.21 (95% CI, 1.15–1.27) for incident risk of IHD per 10 μg/m^3^ increment in long-term PM_2.5_ exposure, while a lower RR of 1.12 (95% CI, 1.07–1.17) was shown after pooling 13 results in men (Supplementary Figures 1, 2). No evidence of publication bias was found either in the funnel plots (Supplementary Figure 3) or by Begg's tests (*P* = 0.33 for women and *P* = 0.73 for men). Further analyses were limited to the 9 studies conducted in both men and women, and the women-to-men RRR for IHD was 1.05 (95% CI, 1.02–1.08) (Figure 2). There was no heterogeneity of between-study observed (*I*^2^=27.1%, *P* = 0.20), and limited publication bias was presented by the funnel plot (Supplementary Figure 4) with Begg's test (*P* = 0.69). The sensitivity analyses showed no substantial changes in the RRRs after excluding the studies one by one.

### Risk of Stroke Associated With PM_2.5_ in Women and Men

A total of 18 articles were included for the association of long-term PM_2.5_ exposure with risk of stroke (Supplementary Table 7). Generally, 11 of the 18 studies defined the outcome using very similar ICD codes, of which 8 studies defined the stroke with the same coding (ICD-9: 430–438; ICD-10: I60–I69) and the other 3 used slightly narrow definitions [i.e., ICD-9: 430–436 (33), ICD-9: 430–437 (19), and ICD-9: 431–438 (27)]. Six studies narrowed the definitions which excluded certain specific codes within the commonly used ICD ranges (ICD-8 or ICD-9: 430–438; ICD-10: I60–I69) (8, 21, 25, 28, 29, 34), and only 1 study did not describe the ICD code (32). Fifteen of the 18 included studies were scored as high-quality.

**Figure 3:**
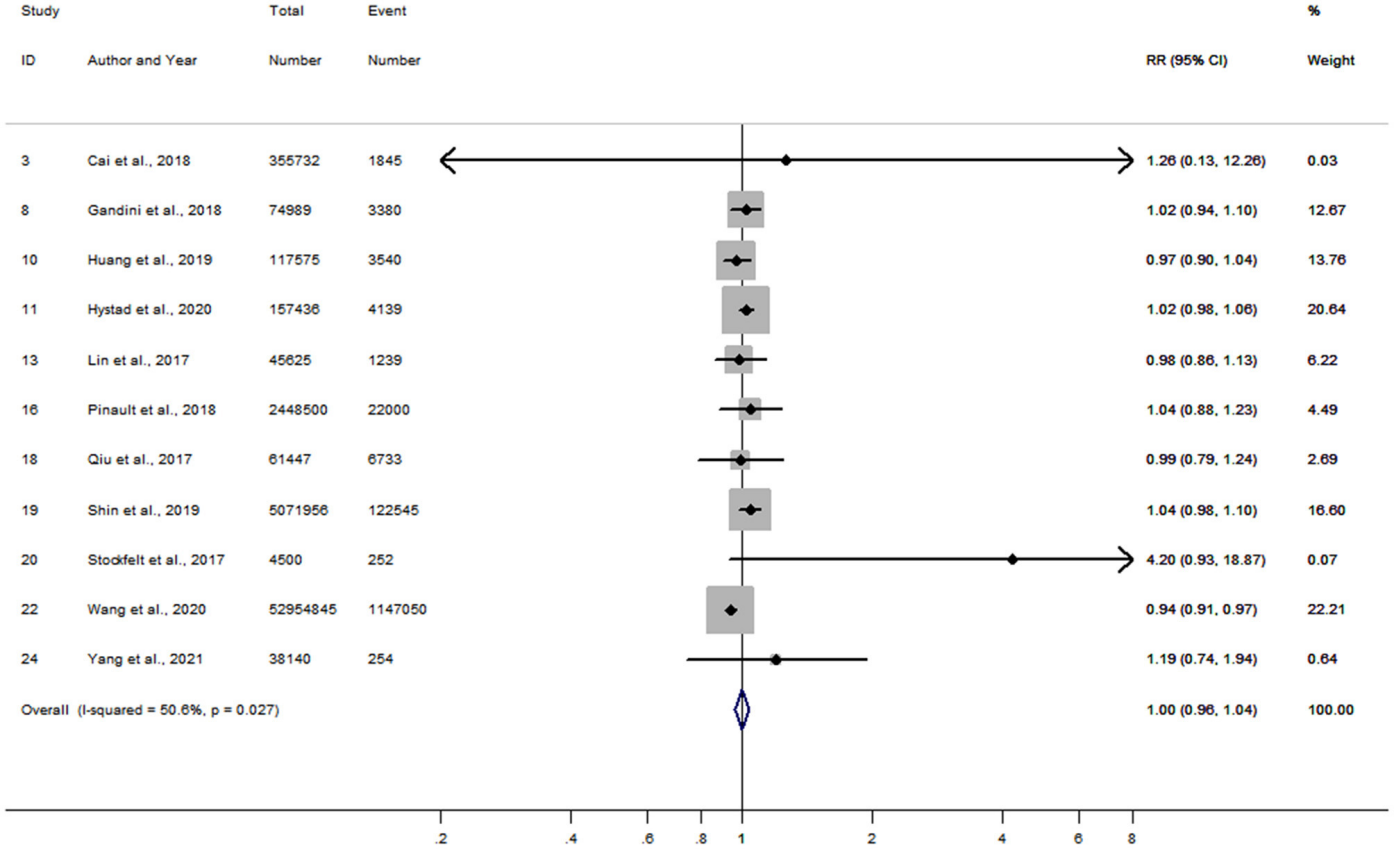
Forest plot for the women-to-men RRR of stroke per 10 μg/m^3^ increase in PM_2.5_ exposure. CI, confidence interval; RRR, ratios of relative risk.

After combining RRs in women reported from 16 studies, a 10 μg/m^3^ increment in long-term PM_2.5_ exposure was associated with an 11% increased risk of stroke (RR = 1.11; 95% CI, 1.08–1.13) (Supplementary Figure 5). The pooled RR in men was also 1.11 (95% CI, 1.07–1.14), similar to that in women (Supplementary Figure 6). A slight publication bias was observed in the analysis of women by the funnel plots (Supplementary Figure 7) with Begg's tests (*P* = 0.05), while no publication bias was found in men (Begg's *P* = 0.22). Moreover, based on 11 articles that reported RRs in women and men within the same study, the combined women-to-men RRR was 1.00 (95% CI, 0.96–1.04) for risk of stroke per 10 μg/m^3^ increase in PM_2.5_ exposure (Figure 3). Heterogeneity of between-study for the analysis was moderate (*I*^2^ = 50.6%, *P* = 0.03), while no publication bias with Begg's test (*P* = 0.14) was observed (Supplementary Figure 8). Sensitivity analysis showed little change on those estimates of RRR after leaving out one study at a time.

## Discussion

The meta-analysis incorporated cohort data of 25 articles among over 36.8 million women and 30.5 million men, which systematically investigated sex-specific associations of long-term PM_2.5_ exposure with risks of IHD and stroke. The meta-analysis obtained a stronger RR for IHD associated with PM_2.5_ exposure in women than that in men. The quantitative estimation of women-to-men RRR indicated that women had a 5% greater risk of IHD per 10 μg/m^3^ increment of PM_2.5_. The associations of long-term PM_2.5_ exposure with stroke were significant in both women and men with similar effect magnitudes between sexes.

Ambient PM_2.5_ pollution has been identified as one of the risk factors contributing to acute cardiac arrest and long-term CVD burden (6, 36). However, it is controversial whether substantial differences would exist in the association of PM_2.5_ exposure with CVD between women and men. Several studies have observed higher risks of CVD or subtype endpoints associated with long-term PM_2.5_ exposure in women (7–9), while others reported similar risks between sexes (10–12). Although a recent meta-analysis has examined effect sizes of long-term exposure to air pollution on the risk of CVD (13), few studies systematically investigated potential sex differences in effect magnitudes for PM_2.5_-CVD association. In this meta-analysis, we extracted data on sex-specific estimations of associations between long-term PM_2.5_ exposure and the mostly reported outcomes of CVD (i.e., IHD and stroke). The pooled women-to-men RRR indicated that women had a 5% higher risk of IHD per 10 μg/m^3^ increase in PM_2.5_. The between-study heterogeneity and publication bias were not observed for the pooled RRR, which enhanced the robustness of the meta-analysis results.

Beyond the novel findings, the design and methods in this meta-analysis had several strengths. First, most of the included studies (21 of 25) were of high quality according to NOS evaluation (Supplementary Table 5), which improved the level of evidence. Second, the data used for the calculation of women-to-men RRR were extracted from the studies that included both men and women. The sex-specific RRs of PM_2.5_ on outcomes were compared in men and women from the same study, which reduced the possibility that potential sex differences were derived from disparities in the background risks of different study populations. Finally, compared to previous meta-analyses searching literature until 2019 (13), one-fourth (7 articles) of the included studies were published after 2019, providing contemporary evidence on sex differences in PM_2.5_-CVD association.

Accumulated studies have documented that sex differences exist in associations of IHD with classic risk factors, such as smoking (4) and diabetes (5), which were more detrimental to women. For instance, one meta-analysis showed that an excess risk of coronary heart disease associated with diabetes existed in women compared with men (5). The present study is the first meta-analysis to identify the significant excess risk of incident IHD associated with long-term exposure to PM_2.5_. The biological mechanisms behind the pooled results are not very clear. One of plausible reasons suggested that pulmonary deposition of inhaled particles under the controlled breathing conditions was found more pronounced in women than in men, which could lead to higher health risk in women (37). Also, studies on personal exposure and biomarkers suggested that women might be more sensitive to inflammatory and oxidative influences of particulate matter (38). Moreover, meta-analyses of epidemiological studies found that PM_2.5_ exposure increased the risk of diabetes (39). It is inferred that diabetes might mediate the sex difference of PM_2.5_-IHD association, considering the evidence on a higher risk of IHD associated with diabetes in women (5). Further epidemiological and experimental researches are needed to explain sex differences in the deleterious impacts of PM_2.5_, and explore specific biological mechanisms involved in PM_2.5_-induced heart disease.

Up to date, although the biological pathways remained unclear, the high-level evidence from meta-analysis reminds us to pay more attention to cardiovascular health in women, when we conduct health risk assessments on air pollution and intervention practices. Accurate health risk assessment is essential to deliver optimal preventive medical care, while it is no longer acceptable to use a one-size-fits-all model of cardiovascular risk stratification which ignores sex differences (40). Many tools or equations of cardiovascular risk assessment widely recommended by guidelines were developed based on sex-specific models along with different effect estimations even for the same risk factor (41, 42). In future studies on air pollution and cardiovascular health, it is encouraged to routinely report sex-specific results of exposure-risk relationship, which may help to accumulate more evidence for risk evaluation and prediction precisely. In the practice of prevention and treatment for IHD, data in US and China showed that women were less likely to be diagnosed appropriately and less frequently receive preventive care, which may be related to a lower perceived risk in women by clinicians and patients (43, 44). Although the knowledge of both health risk from air pollution and measures of CVD prevention should be delivered to everyone, more health education or intervention may be enhanced in women, especially in those middle- and low-income countries where dual challenges of low education in women and heavy air pollution exist (45, 46).

In this meta-analysis, several limitations inherent to the use of the summarized data should be addressed. First, PM_2.5_ is composed of numerous elements, and a recent research has also shown differences in cardiovascular health associations related to different PM_2.5_ components (47). It is unknown whether the sex differences in cardiovascular health are associated with various PM_2.5_ components. Second, PM_2.5_ exposure assessments in the included cohort studies were based on ambient PM_2.5_ levels rather than personal exposure assessment methods, which might ignore the indoor air pollution and result in potential misclassification of exposure. Measurements of personal exposure would be encouraged to obtain more accurate assessment of air pollutant exposure in future studies. Third, temperature extremes may elevate cardiovascular risk independently or jointly with air pollution (35, 48), but the sex-specific effect estimations of long-term PM_2.5_ exposure did not adjust for climate conditions in most of the included studies. Fourth, it is inconsistent for the adjusted covariates in regression models across the original studies. However, most of the studies have adjusted for critical traditional risk factors of CVD, such as age, body mass index (BMI), and smoking. No substantial heterogeneity of between-study in the estimations of RRRs suggested good internal reliability of the meta-analysis results. Last, most of the included articles lacked adjustment for women reproductive factors except that two studies adjusted for the use of oral contraceptives (18) and menopausal status (21). Potential residual confounding may exist due to missing adjustment for reproductive factors in women. More sex-specific quantitative analyses would be encouraged to further validate sex differences in associations of PM_2.5_ exposure on CVD.

## Conclusions

In sum, the meta-analysis provided evidence on the sex-specific risk of CVD associated with long-term PM_2.5_ exposure, and identified a significantly stronger association between PM_2.5_ and risk of IHD in women, compared with men. It suggests to become a routine practice that studies on the association of CVD with air pollution report sex-specific results in the future, which would help to develop evidence-based and sex-specific health policies to reduce disease burden attributable to air pollution.

## Data Availability Statement

The original contributions presented in the study are included in the article/Supplementary Material, further inquiries can be directed to the corresponding author/s.

## Author Contributions

JZ, XW, and XY designed the research. JZ, XW, and MY performed the literature search and extracted and analyzed the data. JZ and XW drafted the initial manuscript. AS, CW, XY, and NT critically reviewed and revised the article. All authors read and approved the submitted version.

## Funding

The work was supported by National Natural Science Foundation of China (grant number 82103928) from the Ministry of Science and Technology of China, and the Fundamental Research Funds for Higher Education of Tianjin Municipal Education Commission (grant number 2021ZD038).

## Conflict of Interest

The authors declare that the research was conducted in the absence of any commercial or financial relationships that could be construed as a potential conflict of interest.

## Publisher's Note

All claims expressed in this article are solely those of the authors and do not necessarily represent those of their affiliated organizations, or those of the publisher, the editors and the reviewers. Any product that may be evaluated in this article, or claim that may be made by its manufacturer, is not guaranteed or endorsed by the publisher.
