# Supplementary material for: Sex Differences in Cardiovascular Risk Associated With Long-Term PM2.5 Exposure: A Systematic Review and Meta-Analysis of Cohort Studies
Source: Front Public Health. 2022 Feb 2;10:802167. doi: 10.3389/fpubh.2022.802167 (PMC8847390; doi:10.3389/fpubh.2022.802167)
Supplement: Supplementary file 1 [file Data_Sheet_1.PDF]

Supplemental Materials

Table S1 PRISMA 2020 Checklist

Section and Topic	Item #	Checklist item	Location where item is reported
**TITLE**			
Title	1	Identify the report as a systematic review.	Page 1
**ABSTRACT**			
Abstract	2	See the PRISMA 2020 for Abstracts checklist.	Page 1
**INTRODUCTION**			
Rationale	3	Describe the rationale for the review in the context of existing knowledge.	Page 2
Objectives	4	Provide an explicit statement of the objective(s) or question(s) the review addresses.	Page 2
**METHODS**			
Eligibility criteria	5	Specify the inclusion and exclusion criteria for the review and how studies were grouped for the syntheses.	Page 2
Information sources	6	Specify all databases, registers, websites, organisations, reference lists and other sources searched or consulted to identify studies. Specify the date when each source was last searched or consulted.	Page 2
Search strategy	7	Present the full search strategies for all databases, registers and websites, including any filters and limits used.	Page 2, and Supplemental Table S2, S3 and S4
Selection process	8	Specify the methods used to decide whether a study met the inclusion criteria of the review, including how many reviewers screened each record and each report retrieved, whether they worked independently, and if applicable, details of automation tools used in the process.	Page 2
Data collection	9	Specify the methods used to collect data from reports, including how many reviewers collected data from each report, whether	Page 2

Section and Topic	Item #	Checklist item	Location where item is reported
process		they worked independently, any processes for obtaining or confirming data from study investigators, and if applicable, details of automation tools used in the process.	
Data items	10a	List and define all outcomes for which data were sought. Specify whether all results that were compatible with each outcome domain in each study were sought (e.g. for all measures, time points, analyses), and if not, the methods used to decide which results to collect.	Page 2
	10b	List and define all other variables for which data were sought (e.g. participant and intervention characteristics, funding sources). Describe any assumptions made about any missing or unclear information.	Page 2
Study risk of bias assessment	11	Specify the methods used to assess risk of bias in the included studies, including details of the tool(s) used, how many reviewers assessed each study and whether they worked independently, and if applicable, details of automation tools used in the process.	Page 2
Effect measures	12	Specify for each outcome the effect measure(s) (e.g. risk ratio, mean difference) used in the synthesis or presentation of results.	Page 2 and 3
Synthesis methods	13a	Describe the processes used to decide which studies were eligible for each synthesis (e.g. tabulating the study intervention characteristics and comparing against the planned groups for each synthesis (item #5)).	Page 2 and 3
	13b	Describe any methods required to prepare the data for presentation or synthesis, such as handling of missing summary statistics, or data conversions.	Page 2 and 3
	13c	Describe any methods used to tabulate or visually display results of individual studies and syntheses.	Page 3
	13d	Describe any methods used to synthesize results and provide a rationale for the choice(s). If meta-analysis was performed, describe the model(s), method(s) to identify the presence and extent of statistical heterogeneity, and software package(s) used.	Page 3
	13e	Describe any methods used to explore possible causes of heterogeneity among study results (e.g. subgroup analysis,	Page 3

Section and Topic	Item #	Checklist item	Location where item is reported
		meta-regression).	
	13f	Describe any sensitivity analyses conducted to assess robustness of the synthesized results.	Page 3
Reporting bias assessment	14	Describe any methods used to assess risk of bias due to missing results in a synthesis (arising from reporting biases).	Page 3
Certainty assessment	15	Describe any methods used to assess certainty (or confidence) in the body of evidence for an outcome.	Page 3
**RESULTS**			
Study selection	16a	Describe the results of the search and selection process, from the number of records identified in the search to the number of studies included in the review, ideally using a flow diagram.	Page 3 and Figure 1
	16b	Cite studies that might appear to meet the inclusion criteria, but which were excluded, and explain why they were excluded.	Figure 1
Study characteristics	17	Cite each included study and present its characteristics.	Page 3-5, Table 1, and Supplemental Table S6 and S7
Risk of bias in studies	18	Present assessments of risk of bias for each included study.	Page 5-6, and Supplemental Table S5
Results of individual studies	19	For all outcomes, present, for each study: (a) summary statistics for each group (where appropriate) and (b) an effect estimate and its precision (e.g. confidence/credible interval), ideally using structured tables or plots.	Page 5-6, and Figure 2-3
Results of syntheses	20a	For each synthesis, briefly summarise the characteristics and risk of bias among contributing studies.	Page 5-6
	20b	Present results of all statistical syntheses conducted. If meta-analysis was done, present for each the summary estimate and its precision (e.g. confidence/credible interval) and measures of statistical heterogeneity. If comparing groups, describe the	Page 5-6, Figure 2 and 3, and

Section and Topic	Item #	Checklist item	Location where item is reported
		direction of the effect.	Supplemental Figure S1, S2, S5, S6
	20c	Present results of all investigations of possible causes of heterogeneity among study results.	Page 5-6
	20d	Present results of all sensitivity analyses conducted to assess the robustness of the synthesized results.	Page 5-6
Reporting biases	21	Present assessments of risk of bias due to missing results (arising from reporting biases) for each synthesis assessed.	Page 5-6, and Supplemental Figure S3, S4, S7, S8
Certainty of evidence	22	Present assessments of certainty (or confidence) in the body of evidence for each outcome assessed.	Page 5-6
**DISCUSSION**			
Discussion	23a	Provide a general interpretation of the results in the context of other evidence.	Page 6
	23b	Discuss any limitations of the evidence included in the review.	Page 7
	23c	Discuss any limitations of the review processes used.	Page 7
	23d	Discuss implications of the results for practice, policy, and future research.	Page 7
**OTHER INFORMATION**			
Registration and protocol	24a	Provide registration information for the review, including register name and registration number, or state that the review was not registered.	Not Registered
	24b	Indicate where the review protocol can be accessed, or state that a protocol was not prepared.	Not prepared
	24c	Describe and explain any amendments to information provided at registration or in the protocol.	NA
Support	25	Describe sources of financial or non-financial support for the review, and the role of the funders or sponsors in the review.	Page 8

Section and Topic	Item #	Checklist item	Location where item is reported
Competing interests	26	Declare any competing interests of review authors.	Page 9
Availability of data, code and other materials	27	Report which of the following are publicly available and where they can be found: template data collection forms; data extracted from included studies; data used for all analyses; analytic code; any other materials used in the review.	All data relevant to the study are included in the article or uploaded as supplementary information.

Table S2. Search strategy for PubMed

No.	Search Query	Results
#1	"particulate matter"[MeSH Terms] OR ("PM2.5"[All Fields] OR "particulate matter"[All Fields] OR "fine particulate matter"[All Fields])	77914
#2	((stroke[MeSH Terms]) OR ("myocardial ischemia" [MeSH Terms]) OR ("coronary artery disease" [MeSH Terms]) OR ("heart failure"[MeSH Terms])) OR (("myocardial infarction"[Title/Abstract]) OR ("ischemic heart disease"[Title/Abstract]) OR ("angina pectoris"[Title/Abstract]) OR ("coronary heart disease"[Title/Abstract]) OR ("heart attack"[Title/Abstract]) OR ("acute coronary syndrome"[Title/Abstract]) OR ("heart failure"[Title/Abstract]) OR (stroke[Title/Abstract]) OR (cerebrovascular disease [Title/Abstract])OR (cardiovascular outcome*[Title/Abstract]) OR (cardiovascular event*[Title/Abstract]) OR (cardiovascular disease*[Title/Abstract]))	1111489
#3	"retrospective studies"[MeSH Terms] OR "longitudinal studies"[MeSH Terms] OR "cohort studies"[MeSH Terms] OR "longitudinal"[All Fields] OR "cohort"[All Fields] OR "odds ratio"[All Fields] OR "relative risk"[All Fields] OR "hazard ratio"[All Fields]	2760342
#4	#1 AND #2 AND #3	1146

Table S3. Search strategy for EMBASE

No.	Search Query	Results
#1	'particulate matter'/exp OR 'particulate matter'	64507
#2	'particulate matter 2.5' OR (fine AND particulate AND matter)	11549
#3	#1 OR #2	64627
#4	'stroke'/exp OR 'stroke':ab,ti OR 'cerebrovascular disease' :ab,ti	518035
#5	'myocardial ischemia'/exp OR 'myocardial infarction':ab,ti OR 'ischemic heart disease':ab,ti OR 'angina pectoris':ab,ti	413668
#6	'coronary artery disease'/exp OR 'coronary heart disease':ab,ti OR 'heart attack':ab,ti OR 'acute coronary syndrome':ab,ti	424136
#7	'heart failure'/exp OR 'heart failure':ab,ti	614831
#8	'cardiovascular outcome*':ab,ti OR 'cardiovascular event*':ab,ti OR 'cardiovascular disease*':ab,ti	326323
#9	#4 OR #5 OR #6 OR #7 OR #8	1819696
#10	'retrospective study'/exp	1069885
#11	'longitudinal study'/exp OR longitudinal	410832
#12	'cohort study'/exp OR cohort OR (odds AND ratio) OR (relative AND risk) OR (hazard AND ratio)	1700761
#13	#10 OR #11 OR #12	2781782
#14	#3 AND #9 AND #13	1062

Table S4. Search strategy for Cochrane Library

No.	Search Query	Results
#1	particulate matter OR PM2.5 OR fine particulate matter	661
#2	stroke OR cerebrovascular disease OR myocardial infarction OR myocardial ischemia OR ischemic heart disease OR coronary heart disease OR coronary artery disease OR heart failure OR angina pectoris OR heart attack OR acute coronary syndrome OR cardiovascular outcome* OR cardiovascular event* OR cardiovascular disease*	202247
#3	retrospective study OR longitudinal study OR cohort study OR longitudinal OR cohort OR odds ratio OR relative risk OR hazard ratio	167354
#4	#1 AND #2 AND #3	32

Table S5. Study quality assessed by Newcastle-Ottawa Assessment Scale

ID	Study (first author and year)	Selection				Comparability	Outcome			Total Score
		Representativeness*	Selection of the non exposed cohort†	Exposure ascertainment‡	No outcome at the start§	Adjust for covariates‖	Outcome assessment#	Follow-up time**	Follow-up adequacy††	
1	Amini et al. (2020)	0	1	1	1	2	1	1	0	7
2	Bai et al. (2019)	1	1	1	1	1	1	1	0	7
3	Cai et al. (2018)	1	1	1	1	2	1	1	1	9
4	Chen et al. (2005)	1	1	1	1	2	1	1	1	9
5	Cramer et al. (2020)	0	1	1	1	2	1	1	0	7
6	Dirgawati et al. (2019)	0	1	1	1	2	1	1	0	7
7	Elliott et al. (2020)	0	1	1	1	2	1	1	1	8
8	Gandini et al. (2018)	1	1	1	0	2	1	0	1	7
9	Hart et al. (2011)	0	1	1	1	1	1	1	0	6
10	Huang et al. (2019)	1	1	1	1	2	1	0	1	8
11	Hystad et al. (2020)	1	1	1	1	2	1	1	0	8
12	Li et al. (2020)	1	1	1	1	2	1	0	1	8
13	Lin et al. (2017)	1	1	1	1	2	0	0	0	6
14	Lipsett et al. (2011)	0	1	1	1	2	1	0	0	6

15	Miller et al. (2007)	0	1	1	1	2	1	0	1	7
16	Pinault et al. (2018)	1	1	1	1	1	1	1	0	7
17	Puett et al. (2011)	0	1	1	1	2	1	1	1	8
18	Qiu et al. (2017)	0	1	1	1	2	1	1	0	7
19	Shin et al. (2019)	1	1	1	1	1	1	1	0	7
20	Stockfelt et al. (2017)	0	1	1	1	2	1	1	0	7
21	Villeneuve et al. (2015)	0	1	1	1	2	1	1	0	7
22	Wang et al. (2020)	0	1	1	1	1	1	0	0	5
23	Wong et al. (2015)	0	1	1	1	2	1	1	0	7
24	Yang et al. (2021)	1	1	1	1	2	1	1	0	8
25	Yin et al. (2017)	0	1	1	1	2	1	1	0	7

[*] Representativeness of the exposed cohort:

1) truly/somewhat representative of the average general population (1 point);

2) selected group of users (e.g. elderly or single-sex cohort) or no description of the derivation of the cohort (0 point).

[†] Selection of the non exposed cohort:

1) drawn from the same community as the exposed cohort (1 point);

2) drawn from a different source or no description of the derivation of the non exposed cohort (0 point).

[‡] Ascertainment of exposure:

1) interpolation model used or data from monitor station (1 point);

2) self report or no description (0 point).

[§] Demonstration that outcome of interest was not present at start of study:

1) yes (1 point);

2) no (0 point).

[‖] Comparability of cohorts on the basis of the design or analysis:

1) study adjusted for age, sex and other covariates (single-sex cohorts were not required to adjust sex) (1 point);

2) study adjusted for smoking, plus age, sex and other covariates (2 points).

[#] Assessment of outcome:

1) independent blind assessment or record linkage (1 point);

2) self report or no description (0 point).

[**]Was follow-up long enough for outcomes to occur:

1) yes (A minimum of 10 years was considered as an adequate follow up period) (1 point);

2) no (0 point).

[††]Adequacy of follow up of cohorts:

1) complete follow up or ≥80% follow-up rate or description provided of those lost (1 point);

2) follow up rate <80% and no description of those lost, or no statement (0 point).

Table S6. Detailed characteristics of eligible study for association of ischemic heart disease with a 10 μg/m^3 increment in $PM_{2.5}$

ID	First author, Year	Study population and Age (range or mean, years)	Exposure measurement methods	ICD coding for IHD	Statistical model and adjusted covariates
2	Bai et al., 2019(1)	long-term residents who lived in Ontario, Canada, from 2001 to 2015<br>Age: 35-85 (53.6±13.2)	GEOS-Chem CTM	ICD-9: 410;<br>ICD-10: I21	Cox model: adjusted for age, sex, urban/rural residency, a north/south indicator, and the four neighborhood level variables (income quintile, percentage of the population 15 years of age and older with less than high school education, unemployment rate, and percentage of recent immigrants)
3	Cai et al., 2018(2)	HUNT study: residents aged ≥20 years in central Norway;<br>EPIC-Oxford: participants aged ≥20 years living throughout the United Kingdom ;<br>UK Biobank: participants aged 40-69 years across the UK<br>Age: ≥20 (52.9±10.6) for all	LUR model	ICD-9: 410-414;<br>ICD-10: I20-I25	Cox model: stratified by sex, with age as the underlying time-scale; adjusted for cohort, education level (low, medium, high), employment (yes or no) and smoking status (never-, ex- and current-)
4	Chen et al., 2005(3)	nonsmoking, non-Hispanic whites<br>Age: ≥25	daily measures of visibility using regression equations relating $PM_{2.5}$ and visibility	ICD-9: 410-414	Sex-specific, time-varying Cox model with attained age as the time variable: adjusted for smoking status, years of education, body mass index (BMI), meat consumption, calendar time by adding the sandwich variance estimate to adjust for correlated observations within each airshed
5	Cramer et al., 2020(4)	female nurses<br>Age: >44	Danish air pollution modeling system (DEHM/UBM/AirGIS)	ICD-8: 410;<br>ICD-10: I21	Time-varying Cox model: adjusted for age (underlying time), year of cohort entry (1993 or 1999), smoking status, pack-years, consumption of fruit, avoidance of fatty meat, alcohol consumption, use of oral contraceptives, use of hormone therapy, physical activity, marital status, employment status, level of urbanization, L_{den}

7	Elliott et al., 2020(5)	U.S. female registered nurses<br>Age: 30-55 (63.1±8.9)	spatiotemporal model	ICD-9: 410	Time-varying Cox model: adjusted for age and race (White yes/no); incident cancer (yes/no), family history of myocardial infarction (yes/no), smoking status (never, past, current), pack-years, Alternate Healthy Eating Index score quartiles, alcohol consumption (0.0, <5.0, 5.0-9.9, 10.0-19.9, or ≥20.0g/d), multivitamin use (yes/no), census tract median income (USD), census tract median home value (USD), occupation father (professional or other), occupation mother (housewife or other), husband's level of education more than high school (yes/no), registered nursing degree in 1992 (yes/no), marital status (married or not married), retirement status (retired or not retired)
9	Hart et al., 2011(6)	men in the U.S. trucking industry<br>Age: 15.3-84.9 (42.1±9.9)	monitor	ICD-9: 410-414; ICD-10: I20-25	Cox model: generated using attained age in 1-year increments as the timeline, with separate baseline hazards based on decade of age at entry, calendar year and decade of hire, and adjusted for race, Census region of residence, the healthy worker survivor effect, and years of work in each of the eight job groups
11	Hystad et al., 2020(7)	general population<br>Age: 35-70 (50.2±9.7)	Satelite + GEOS-Chem + monitor	ICD-10: I21-I22	Cox frailty model: adjusted for age, sex, baseline year, community random effect, smoking status, physical activity, PURE diet index score, waist-to hip ratio, INTERHEART risk score, use of solid fuels for cooking, education level, household wealth index, occupational class, baseline cardiovascular disease and chronic conditions, cardiovascular disease medication use, and hypertension (determined a priori), geographical covariates (urban or rural location, baseline country gross domestic product per capita, Night Light Development Index score, and a national or regional Healthcare Access and Quality Index)
12	Li et al., 2020(8)	general population<br>Age: ≥18 (50.98±11.86)	satellite-based spatiotemporal model	NA	Cox model: adjusted for age, sex, geographical region, urbanization, education level, smoking, drinking, work-related physical activity, body mass index, systolic blood pressure, serum glucose and total cholesterol
14	Lipsett et al., 2011(9)	female public school professionals<br>Age: 22-104 (median, 53)	monitoring interpolation	ICD-9: 410-414; ICD-10: I20-I25	Cox model: adjusted for age, race, smoking status, total pack-years, body mass index, marital status, alcohol consumption, second-hand smoke exposure at home, dietary fat, dietary fiber, dietary calories, physical activity, menopausal status, hormone therapy use, family history of MI or stroke, blood pressure medication, and aspirin use, and for contextual variables (income, income inequality, education, population size, racial composition, and unemployment)

15	Miller et al., 2007(10)	postmenopausal women<br>Age: 50-79 (median, 63)	monitoring	NA	Cox model: adjusted for age, race or ethnic group, educational level, household income, smoking status, systolic blood pressure, body-mass index, and presence or absence of diabetes, hypertension, or hypercholesterolemia
16	Pinault et al., 2018(11)	noninstitutional population in Canada<br>Age: 25-90	Satelite + GEOS-Chem + GWR surface monitor	ICD-10: I20-I25	Cox model: stratified by age (5-year categories), sex, airshed, and population centre size and adjusted for visible minority status, Aboriginal identity, marital status, educational attainment, income quintile, labour force status, and Census Division (CD)-level ecological covariates
17	Puett et al., 2011(12)	male dentists, pharmacists, optometrists, podiatrists, osteopaths, and veterinarians<br>Age: 40-75 (57.4±9.8)	Spatiotemporal models: GIS-based spatial smoothing model	NA	Time-varying Cox model: stratified by age in months, adjusting for year, season, state of residence, body mass index (BMI), hypertension, hypercholesterolemia, diabetes, family history of MI, smoking (status and pack-years), physical activity, healthy diet, and alcohol consumption
20	Stockfelt et al., 2017(13)	Primary Prevention Study (PPS) cohort: general male residents<br>Age: 64-75 (mean, 69)	Dispersion model	ICD-9: 410-414; ICD-10: I20-I25	Cox model: adjusted for age (as time scale), smoking class, occupational class, marital status, leisure-time physical activity, calendar year, and mean income in the Small Areas for Market Statistics (SAMS) area
		GOT-MONICA cohort: all residents in Gothenburg aged 25-64 years<br>Age: 25-64 (mean, 46)	Dispersion model	ICD-9: 410-414; ICD-10: I20-I25	Cox model: adjusted for age (as time scale), smoking class, marital status, leisure-time physical activity, calendar year, mean income in the Small Areas for Market Statistics (SAMS) area, sex and enrollment year
21	Villeneuve et al., 2015(14)	general female population<br>Age: 40-59 (48.5±5.6)	Satellite + GEOS-Chem	ICD-9: 410-414; ICD-10: I20-I25	Cox model: adjusted for age at entry, body mass index, cigarette smoking, occupation, marital status, attained education, and four contextual variables derived from census area measures (mean income, proportion with high school education, percentage of low income households, and unemployment rate)
22	Wang et al., 2020(15)	Medicare beneficiaries living in the US between 2000 and 2008<br>Age: 65-120	spatiotemporal models	ICD-10: I20-I25	Cox model: strata for age, sex, race, and ZIP code and controlled for neighborhood socio-economic status (SES)
23	Wong et al., 2015(16)	general elder participants<br>Age: ≥65 (mean, 71.9)	Satellite-based model	ICD-10: I20-I25	Cox model: adjusted for age, sex, body mass index, smoking, physical exercise, education, monthly expenses, the Tertiary Planning Units (TPU)-level proportion of the population ≥

					65 years of age, the proportion with > secondary education, the average monthly income in each TPU and the proportion of smokers in each district
25	Yin et al., 2017(17)	general male participants recruited during 1990-1991 from 45 areas in China<br>Age: ≥40 (54.8±10.7)	Satelite + GEOS-Chem + surface monitor	ICD-9: 410-414	Cox model: adjusted for age, individual-level covariates including marital status, educational level, smoking status, years of smoking, cigarettes per day, passive smoking, occupational exposure, alcohol drinking, units of alcohol per week, body mass index (BMI), consumption of fresh fruits and vegetables, household solid-fuel use and area-level covariates including urban/rural, region, and mean years of education

Table S7. Detailed characteristics of eligible study for association of stroke with a 10 μg/m^3 increment in $PM_{2.5}$

ID	First author, Year	Study population and Age (range or mean, years)	Exposure measurement methods	ICD coding for stroke	Statistical model and adjusted covariates
1	Amini et al., 2020(18)	female nurses<br>Age: >44 (52.6 ±7.7)	Danish air pollution modeling system (DEHM/UBM/AirGIS)	ICD-8: 431,432, 433, 434, 436;<br>ICD-10: I61, I63, I64	Time-varying Cox model: adjusted for age as underlying timeline, year of entry to cohort (1993 or 1999), calendar year, income, degree of urbanicity, physical activity, alcohol drinking, smoking, marital status, fruit consumption, noise
3	Cai et al., 2018(2)	HUNT study: residents aged ≥20 years in central Norway;<br>EPIC-Oxford: participants aged ≥20 years living throughout the United Kingdom ;<br>UK Biobank: participants aged 40-69 years across the UK<br>Age: ≥20 (52.9±10.6) for all	LUR model	ICD-9: 430-438;<br>ICD-10: I60-I69	Cox model: stratified by sex, with age as the underlying time-scale; adjusted for cohort, education level (low, medium, high), employment (yes or no) and smoking status (never-, ex- and current-)
6	Dirgawati et al., 2019(19)	general elder men in Australia<br>Age: ≥65 (72.1±4.4)	LUR model	ICD-9: 430, 431, 433.x1, 434.x1, 436;<br>ICD-10: I60, I61, I63, I64	Cox model: adjusted for age, smoking history (never-smokers, former-smokers who had quit ≥10 years, former-smokers who had quit <10 years, current-smokers), and tobacco consumption among current smokers (g/day), body mass index (BMI), and history of hypertension

7	Elliott et al., 2020(5)	U.S. female registered nurses<br>Age: 30-55 (63.1±8.9)	spatiotemporal model	ICD-9: 430-437	Time-varying Cox model: adjusted for age and race (White yes/no); incident cancer (yes/no), family history of myocardial infarction (yes/no), smoking status (never, past, current), pack-years, Alternate Healthy Eating Index score quartiles, alcohol consumption, multivitamin use (yes/no), census tract median income (USD), census tract median home value (USD), occupation father (professional or other), occupation mother (housewife or other), husband's level of education more than high school (yes/no), registered nursing degree in 1992 (yes/no), marital status (married or not married), retirement status (retired or not retired)
8	Gandini et al., 2018(20)	general population<br>Age: >35	the modeling system MINNI: CTM + meteorological prognostic fields	ICD-9: 430-438	Cox model: adjusted by age, educational level, living with partner, occupational status, smoking habit, physical activity status, type of municipality (rural, urban, metropolitan areas) and body mass index (BMI)
10	Huang et al., 2019(21)	general population<br>Age: ≥18 (50.9±11.8)	satellite based spatiotemporal model	ICD-10: I60-I69	Cox model: adjusted for age, sex, geographic region, urbanization; education, smoking status, alcohol consumption, physical activity, body mass index, and hypertension
11	Hystad et al., 2020(7)	general population<br>Age: 35-70 (50.2±9.7)	Satelite + GEOS-Chem + monitor	ICD-10: I60-I64, I69	Cox frailty model: adjusted for age, sex, baseline year, community random effect, smoking status, physical activity, PURE diet index score, waist-to hip ratio, INTERHEART risk score, use of solid fuels for cooking, education level, household wealth index, occupational class, baseline cardiovascular disease and chronic conditions, cardiovascular disease medication use, and hypertension (determined a priori), geographical covariates (urban or rural location, baseline country gross domestic product per capita, Night Light Development Index score, and a national or regional Healthcare Access and Quality Index)

13	Lin et al., 2017(22)	general population<br>Age: ≥18 (58.3±14.7)	Satelite + GEOS-Chem	NA	Multilevel logistic regression model: adjusted for age, sex, body mass index, consumption of fruit and vegetables, smoking, alcohol drinking, marital status, urbanity, household income, education, fuel type, ventilation, hypertension status, and treatment
14	Lipsett et al., 2011(9)	female public school professionals<br>Age: 22-104 (median, 53)	monitoring interpolation	ICD-9: 431-434,436<br>ICD-10: I61-I64	Cox model: adjusted for age, race, smoking status, total pack-years, body mass index, marital status, alcohol consumption, second-hand smoke exposure at home, dietary fat, dietary fiber, dietary calories, physical activity, menopausal status, hormone therapy use, family history of MI or stroke, blood pressure medication, and aspirin use, and for contextual variables (income, income inequality, education, population size, racial composition, and unemployment)
15	Miller et al., 2007(10)	postmenopausal women<br>Age: 50-79 (median, 63)	monitoring	ICD-9-CM: 430-438	Cox model: adjusted for age, race or ethnic group, educational level, household income, smoking status, systolic blood pressure, body-mass index, and presence or absence of diabetes, hypertension, or hypercholesterolemia
16	Pinault et al., 2018(11)	noninstitutional population in Canada<br>Age: 25-90	Satelite + GEOS-Chem + GWR surface monitor	ICD-10: I60-I69	Cox model: stratified by age (6-year categories), sex, airshed, and population centre size and adjusted for visible minority status, Aboriginal identity, marital status, educational attainment, income quintile, labour force status, and Census Division (CD)-level ecological covariates
18	Qiu et al., 2017(23)	general elder population<br>Age: ≥65 (mean, 72)	satellite-based model	ICD-9: 430-436	Cox model: adjusted for age, sex, body mass index, physical exercise, education, monthly expenses, smoking status, alcohol drinking, medication taken, the tertiary planning units level covariates (prevalence of age ≥65 years, tertiary education, and income ≥$1,923/mo USD), and smoking rate at district level

19	Shin et al., 2019(24)	long-term residents of Ontario<br>Age: 35-85 (53.2±12.9)	satellite-derived data + LUR model	ICD-9: 434, 436, 430, 431; ICD-10: I63.x [excluding I63.6], I64, H34.1, I60, I61	Cox model: stratified by an indicator for living in the Greater Toronto Area or not, and adjusted for age, sex, area-level socioeconomic status (education, recent immigrants, unemployment rate, and income quintile), urban/rural area, and northern/southern Ontario
20	Stockfelt et al., 2017(13)	Primary Prevention Study (PPS) cohort: general male residents<br>Age: 64-75 (mean, 69)	Dispersion model	ICD-9: 431-436; ICD-10: I61-I65	Cox model: adjusted for age (as time scale), smoking class, occupational class, marital status, leisure-time physical activity, calendar year, and mean income in the Small Areas for Market Statistics (SAMS) area
		Primary Prevention Study (PPS) cohort: general male residents<br>Age: 64-75 (mean, 69)	Dispersion model	ICD-9: 431-436; ICD-10: I61-I65	Cox model: adjusted for age (as time scale), smoking class, marital status, leisure-time physical activity, calendar year, mean income in the Small Areas for Market Statistics (SAMS) area, sex and enrollment year
21	Villeneuve et al., 2015(14)	general female population<br>Age: 40-59 (48.5±5.6)	Satellite + GEOS-Chem	ICD-9: 430-438; ICD-10: I60-I69	Cox model: adjusted for age at entry, body mass index, cigarette smoking, occupation, marital status, attained education, and four contextual variables derived from census area measures (mean income, proportion with high school education, percentage of low income households, and unemployment rate)
22	Wang et al., 2020(15)	Medicare beneficiaries living in the US between 2000 and 2008<br>Age: 65-120	spatiotemporal models	ICD-10: I60-I69	Cox model: strata for age, sex, race, and ZIP code and controlled for neighborhood socio-economic status (SES)
24	Yang et al., 2021(25)	general population<br>Age: ≥22 (43.96±13.68)	satellite-based spatiotemporal model	ICD-10: I60-I69	Time-varying Cox model: adjusted for age, sex, education, personal monthly income (<500 Yuan vs ≥ 500 Yuan); body mass index (BMI), smoke (yes vs no), drink (yes vs no), physical activity (inactive vs active), history of hypertension (yes vs no) and diabetes (yes vs no)

25	Yin et al., 2017(17)	general male participants recruited during 1990-1991 from 45 areas in China<br>Age: ≥40 (54.8±10.7)	Satelite + GEOS-Chem + surface monitor	ICD-9: 431-438	Cox model: adjusted for age, individual-level covariates including marital status, educational level, smoking status, years of smoking, cigarettes per day, passive smoking, occupational exposure, alcohol drinking, units of alcohol per week, body mass index (BMI), consumption of fresh fruits and vegetables, household solid-fuel use and area-level covariates including urban/rural, region, and mean years of education

Figure S1. Forest plot for the risk of ischemic heart disease per 10 μg/m^3 increment in long-term $PM_{2.5}$ exposure in women.

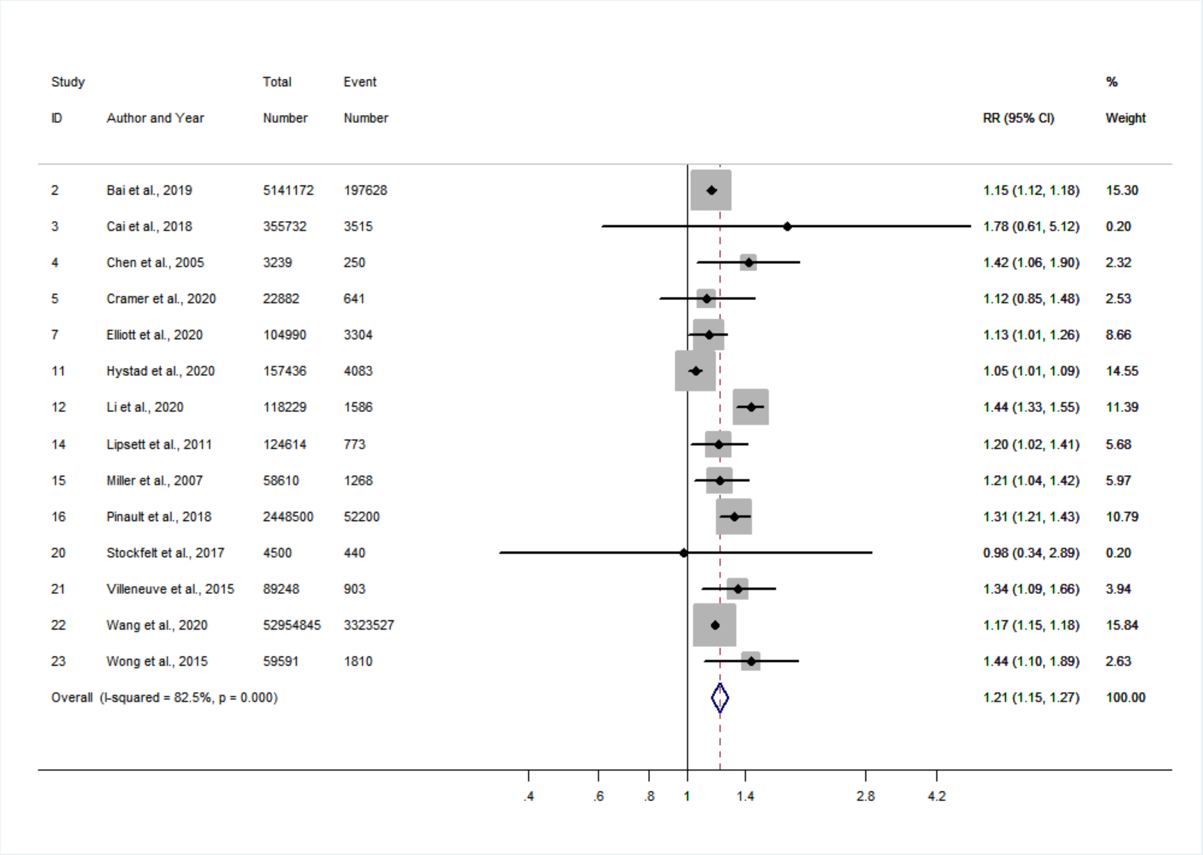

Figure S2. Forest plot for the risk of ischemic heart disease per 10 μg/m^3 increment in long-term $PM_{2.5}$ exposure in men.

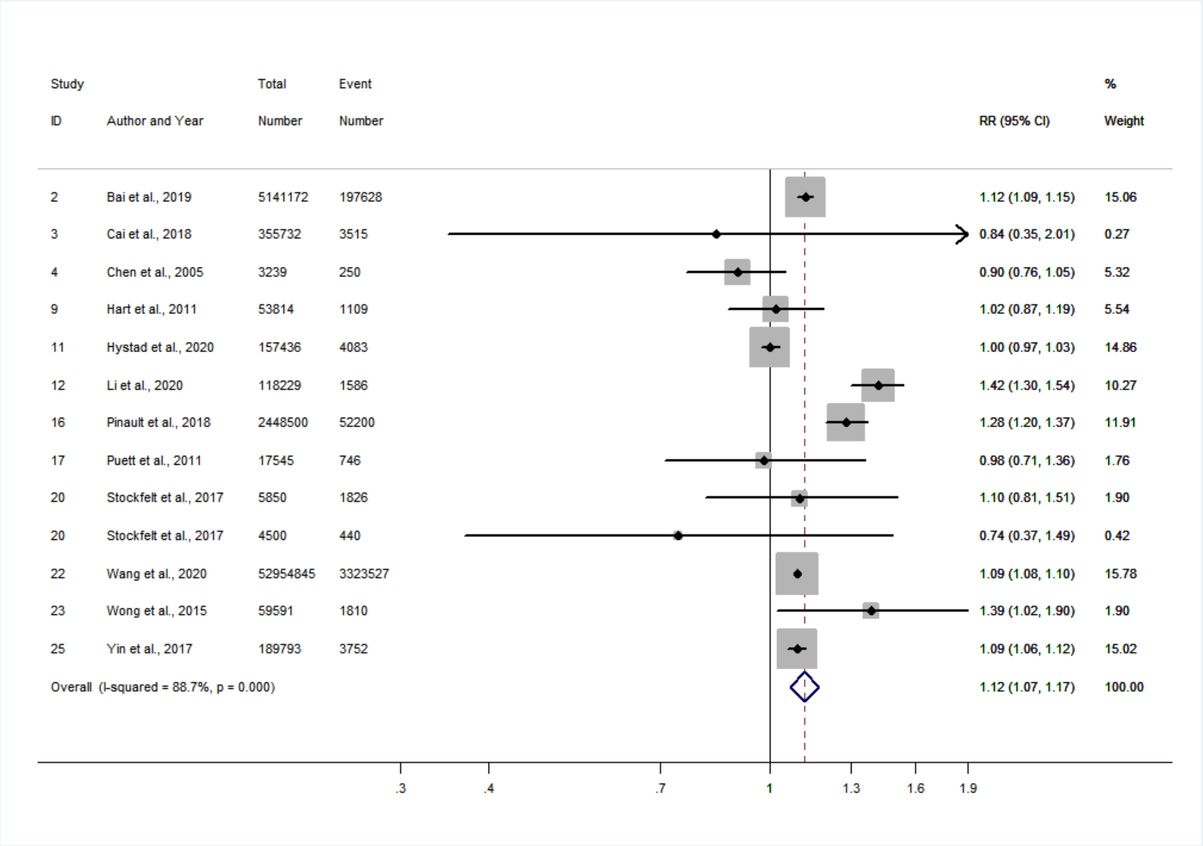

Figure S3. Funnel plots for relative risk (RR) of ischemic heart disease in women (A) and men (B).

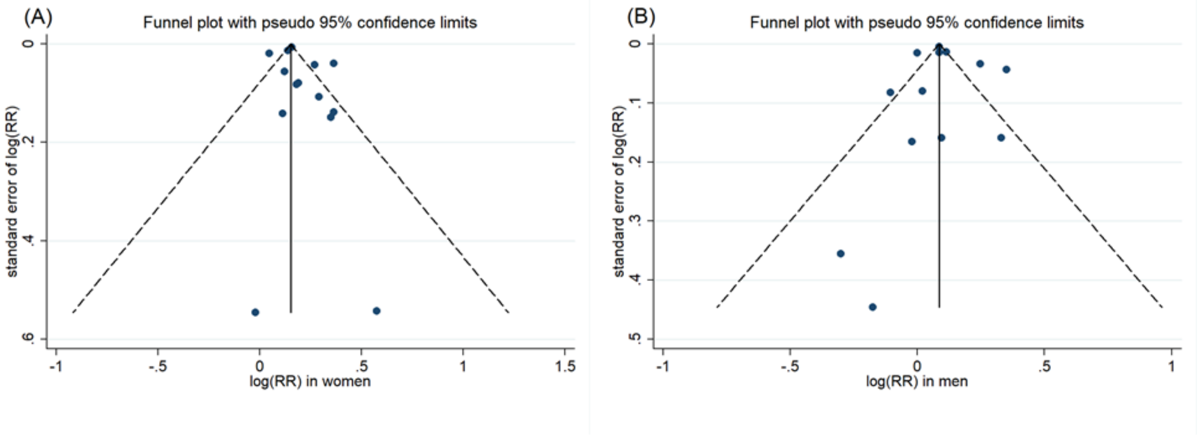

Figure S4. Funnel plots for the women-to-men ratios of relative risk (RRR) of ischemic heart disease.

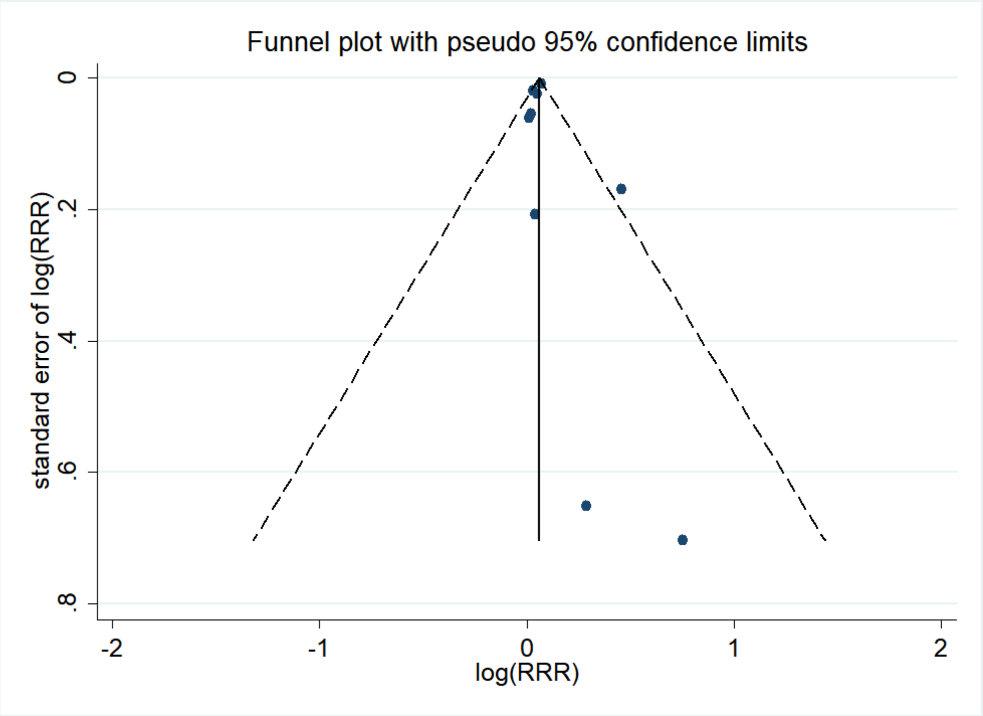

Figure S5. Forest plot for the risk of stroke per 10 μg/m^3 increment in long-term $PM_{2.5}$ exposure in women.

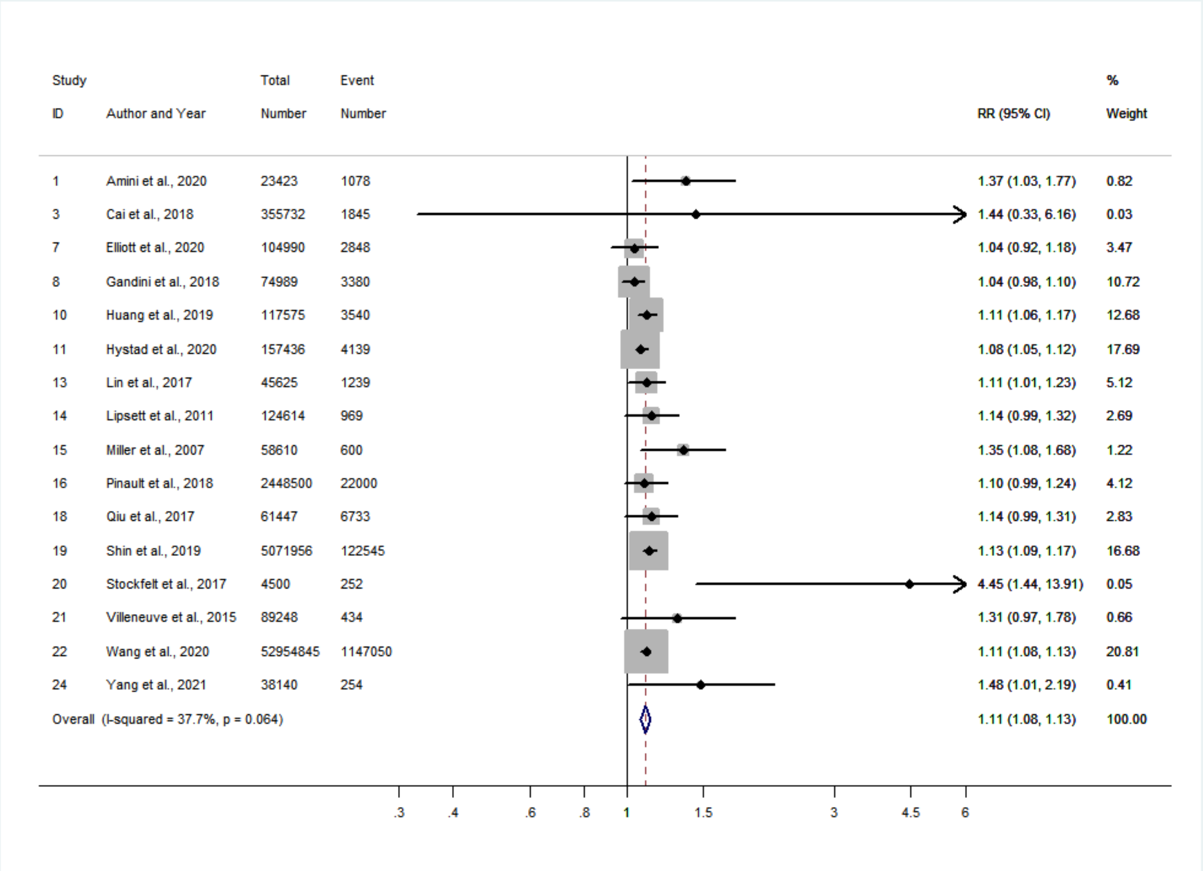

Figure S6. Forest plot for the risk of stroke per 10 μg/m^3 increment in long-term $PM_{2.5}$ exposure in men.

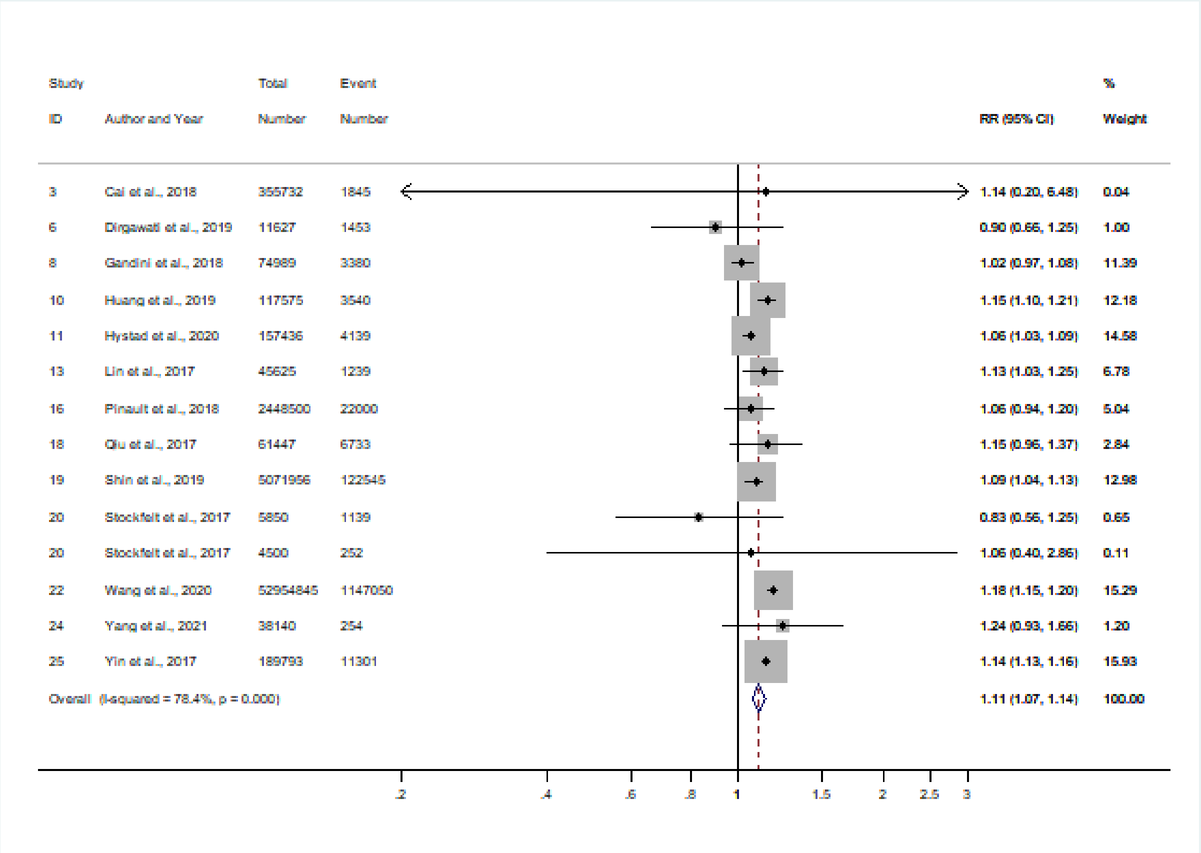

Figure S7. Funnel plots for relative risk (RR) of stroke in women (A) and men (B).

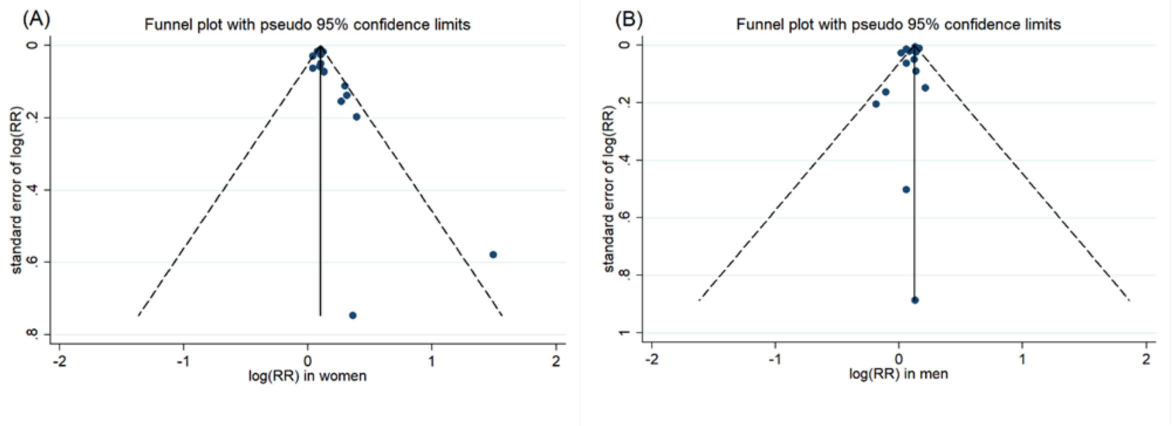

Figure S8. Funnel plots for the women-to-men ratios of relative risk (RRR) of stroke.

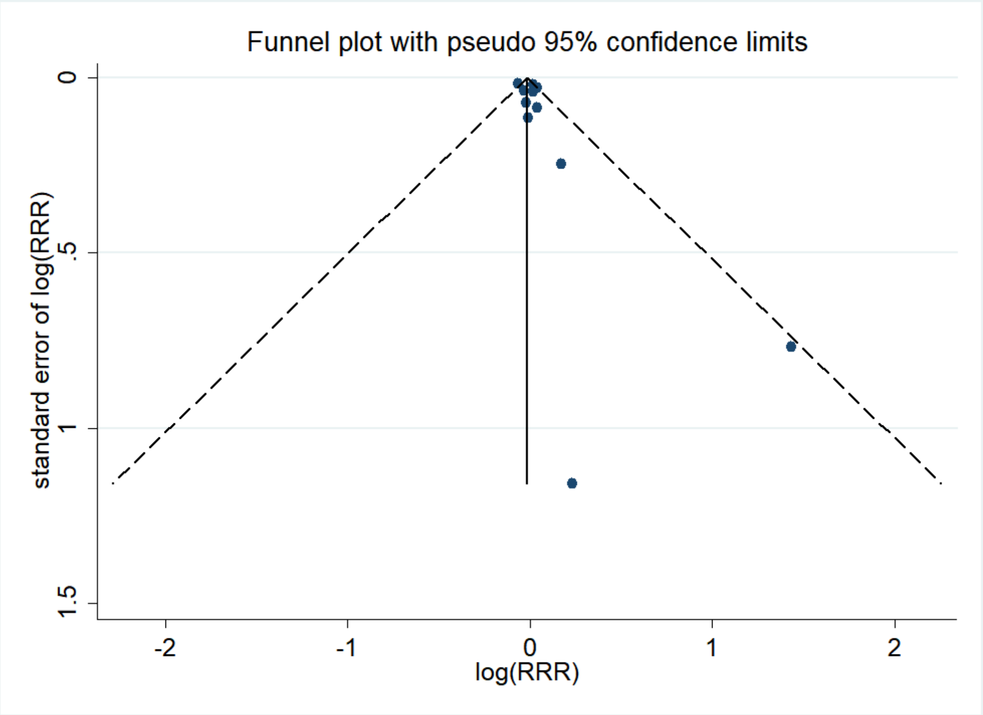

Supplemental Reference

1. Bai L, Shin S, Burnett RT et al. Exposure to ambient air pollution and the incidence of congestive heart failure and acute myocardial infarction: A population-based study of 5.1 million Canadian adults living in Ontario. Environment international 2019;132:105004.
2. Cai Y, Hodgson S, Blangiardo M et al. Road traffic noise, air pollution and incident cardiovascular disease: A joint analysis of the HUNT, EPIC-Oxford and UK Biobank cohorts. Environment international 2018;114:191-201.
3. Chen LH, Knutsen SF, Shavlik D et al. The association between fatal coronary heart disease and ambient particulate air pollution: Are females at greater risk? Environmental health perspectives 2005;113:1723-9.
4. Cramer J, Jørgensen JT, Hoffmann B et al. Long-Term Exposure to Air Pollution and Incidence of Myocardial Infarction: A Danish Nurse Cohort Study. Environmental health perspectives 2020;128:57003.
5. Elliott EG, Laden F, James P, Rimm EB, Rexrode KM, Hart JE. Interaction between Long-Term Exposure to Fine Particulate Matter and Physical Activity, and Risk of Cardiovascular Disease and Overall Mortality in U.S. Women. Environmental health perspectives 2020;128:127012.
6. Hart JE, Garshick E, Dockery DW, Smith TJ, Ryan L, Laden F. Long-term ambient multipollutant exposures and mortality. Am J Respir Crit Care Med 2011;183:73-8.
7. Hystad P, Larkin A, Rangarajan S et al. Associations of outdoor fine particulate air pollution and cardiovascular disease in 157 436 individuals from 21 high-income, middle-income, and low-income countries (PURE): a prospective cohort study. The Lancet Planetary health 2020;4:e235-e245.
8. Li J, Liu F, Liang F et al. Long-Term Effects of High Exposure to Ambient Fine Particulate Matter on Coronary Heart Disease Incidence: A Population-Based Chinese Cohort Study. Environmental science & technology 2020;54:6812-6821.
9. Lipsett MJ, Ostro BD, Reynolds P et al. Long-term exposure to air pollution and cardiorespiratory disease in the California teachers study cohort. Am J Respir Crit Care Med 2011;184:828-35.
10. Miller KA, Siscovick DS, Sheppard L et al. Long-term exposure to air pollution and incidence of cardiovascular events in women. The New England journal of medicine 2007;356:447-58.
11. Pinault L, Brauer M, Crouse DL et al. Diabetes Status and Susceptibility to the Effects of PM2.5 Exposure on Cardiovascular Mortality in a National Canadian Cohort. Epidemiology (Cambridge, Mass) 2018;29:784-794.
12. Puett RC, Hart JE, Suh H, Mittleman M, Laden F. Particulate matter exposures, mortality, and cardiovascular disease in the health professionals follow-up study. Environmental health perspectives 2011;119:1130-5.
13. Stockfelt L, Andersson EM, Molnár P et al. Long-term effects of total and source-specific particulate air pollution on incident cardiovascular disease in Gothenburg, Sweden. Environmental research 2017;158:61-71.
14. Villeneuve PJ, Weichenthal SA, Crouse D et al. Long-term Exposure to Fine Particulate Matter Air Pollution and Mortality among Canadian Women.

Epidemiology (Cambridge, Mass) 2015;26:536-545.
15. Wang B, Eum KD, Kazemiparkouhi F et al. The impact of long-term PM2.5 exposure on specific causes of death: Exposure-response curves and effect modification among 53 million U.S. Medicare beneficiaries. Environmental Health: A Global Access Science Source 2020;19.
16. Wong CM, Lai HK, Tsang H et al. Satellite-Based Estimates of Long-Term Exposure to Fine Particles and Association with Mortality in Elderly Hong Kong Residents. Environmental health perspectives 2015;123:1167-72.
17. Yin P, Brauer M, Cohen A et al. Long-term Fine Particulate Matter Exposure and Nonaccidental and Cause-specific Mortality in a Large National Cohort of Chinese Men. Environmental health perspectives 2017;125:117002.
18. Amini H, Dehlendorff C, Lim YH et al. Long-term exposure to air pollution and stroke incidence: A Danish Nurse cohort study. Environment international 2020;142:105891.
19. Dirgawati M, Hinwood A, Nedkoff L et al. Long-term Exposure to Low Air Pollutant Concentrations and the Relationship with All-Cause Mortality and Stroke in Older Men. Epidemiology (Cambridge, Mass) 2019;30 Suppl 1:S82-s89.
20. Gandini M, Scarinzi C, Bande S et al. Long term effect of air pollution on incident hospital admissions: Results from the Italian Longitudinal Study within LIFE MED HISS project. Environment international 2018;121:1087-1097.
21. Huang K, Liang F, Yang X et al. Long term exposure to ambient fine particulate matter and incidence of stroke: prospective cohort study from the China-PAR project. BMJ (Clinical research ed) 2019;367:l6720.
22. Lin H, Guo Y, Di Q et al. Ambient PM(2.5) and Stroke: Effect Modifiers and Population Attributable Risk in Six Low- and Middle-Income Countries. Stroke 2017;48:1191-1197.
23. Qiu H, Sun S, Tsang H et al. Fine particulate matter exposure and incidence of stroke: A cohort study in Hong Kong. Neurology 2017;88:1709-1717.
24. Shin S, Burnett RT, Kwong JC et al. Ambient Air Pollution and the Risk of Atrial Fibrillation and Stroke: A Population-Based Cohort Study. Environmental health perspectives 2019;127:87009.
25. Yang X, Zhang L, Chen X et al. Long-term exposure to ambient PM(2.5) and stroke mortality among urban residents in northern China. Ecotoxicology and environmental safety 2021;213:112063.
